# Supplementary material for: Molecular characterization of carbapenem-resistant Acinetobacter baumannii clinical isolates from Egyptian patients
Source: PLoS One. 2021 Jun 24;16(6):e0251508. doi: 10.1371/journal.pone.0251508 (PMC8224909; doi:10.1371/journal.pone.0251508)
Supplement: Attachment — Submitted filename: Response to Reviewers.docx [file pone.0251508.s004.docx]

1. Level 1 heading in 18pt font and level 2 in 16pt font
2. Table not cited in bold, table title above it and not below
3. Reem M Hassan^1^ and Yasmine S Elkholy^3^ edited our manuscript.
4. Being teaching hospital, before admission every patient gives an informed consent. Samples were collected for diagnostic purposes and were furtherly investigated for better analysis of antimicrobial resistance to allow control.
5. We used retained strains as controls, sample size was calculated as 190.

Using proportion (one sample) for sample size calculation in Stata (version 16), a reference proportion of (61.5%) was used (Nevine Fam et al., 2019). A mean proportion of 72% was used based on the studies for Egyptian population with bla oxa-23-like as the most prevalent trait (El Bannah AMS et al., 2017). Alpha was set at 0.05 with 80% power. The calculated sample size is 190.

6. All data underlying the findings are fully available.
7. Yes, you can publish the peer review history of our article.
8. Result section has been moved after material and methods.
9. The prefix bla has been italicized
10. In discussion, I compared results with data from Algeria, Palestine, middle east, and Europe.
